# Proteases of haematophagous arthropod vectors are involved in blood-feeding, yolk formation and immunity - a review

**DOI:** 10.1186/s13071-017-2005-z

**Published:** 2017-02-13

**Authors:** Paula Beatriz Santiago, Carla Nunes de Araújo, Flávia Nader Motta, Yanna Reis Praça, Sébastien Charneau, Izabela M. Dourado Bastos, Jaime M. Santana

**Affiliations:** 1 Laboratório de Interação Patógeno-Hospedeiro, Departamento de Biologia Celular, Instituto de Ciências Biológicas, Universidade de Brasília, Campus Universitário Darcy Ribeiro, Asa Norte, 70910-900 Brasília, DF Brazil; 2 Faculdade de Ceilândia, Universidade de Brasília, Centro Metropolitano, Conjunto A, Lote 01, 72220-275 Brasília, DF Brazil; 3 Programa Pós-Graduação em Ciências Médicas, Faculdade de Medicina, Universidade de Brasília, Campus Universitário Darcy Ribeiro, Asa Norte, 70910-900 Brasília, DF Brazil; 4 Laboratório de Bioquímica e Química de Proteínas, Departamento de Biologia Celular, Instituto de Ciências Biológicas, Universidade de Brasília, Campus Universitário Darcy Ribeiro, Asa Norte, 70910-900 Brasília, DF Brazil

**Keywords:** Proteases, Haematophagy, Digestion, Yolk formation, Immunity, Ticks, Triatomines, Mosquitoes

## Abstract

Ticks, triatomines, mosquitoes and sand flies comprise a large number of haematophagous arthropods considered vectors of human infectious diseases. While consuming blood to obtain the nutrients necessary to carry on life functions, these insects can transmit pathogenic microorganisms to the vertebrate host. Among the molecules related to the blood-feeding habit, proteases play an essential role. In this review, we provide a panorama of proteases from arthropod vectors involved in haematophagy, in digestion, in egg development and in immunity. As these molecules act in central biological processes, proteases from haematophagous vectors of infectious diseases may influence vector competence to transmit pathogens to their prey, and thus could be valuable targets for vectorial control.

**Electronic supplementary material:**

The online version of this article (doi:10.1186/s13071-017-2005-z) contains supplementary material, which is available to authorized users.

## Background

Haematophagous arthropod vectors are spread worldwide. They are of medical and veterinary importance since their blood-feeding habit provides a scenario for the transmission of a variety of pathogens, including virus, bacteria, protozoans and helminths [1]. Although there are clinical differences among the diseases caused by these organisms, they share the tendency to coexist in low and middle-income countries. Additionally, for most of the infectious diseases transmitted by invertebrate vectors there are neither vaccines nor preventive treatments. Few chemotherapy drugs are available for the treatment with many serious adverse reactions and rapid emergence of resistant strains, generating social and economic losses in those countries. Chikungunya, Mayaro and Zika virus infections, Crimean-Congo haemorrhagic fever, dengue fever, Japanese encephalitis, Rift Valley fever, tick-borne encephalitis, West Nile fever, yellow fever, Lyme disease, plague, rickettsiosis, tularaemia, Chagas disease, leishmaniasis, malaria, sleeping sickness, lymphatic filariasis and onchocerciasis are all examples of vector-borne diseases with global impact on morbidity and mortality (Table 1) since they affect more than one billion individuals and cause over one million deaths every year [2].

**Table 1:** Vector-borne diseases

Disease	Pathogen	Estimated number	Major distribution	Major vectors
Chikungunya	Chikungunya virus: *Alphavirus* (Togaviridae)	37,480 (Americas, 2015)	Africa, the Americas, Asia, Europe	*Aedes* spp.
Mayaro fever	Mayaro virus: *Alphavirus* (Togaviridae)	197 cases (2015)^a^	South America	*Haemagogus janthinomys*
Zika	Zika virus: *Flavivirus* (Flaviviridae)	No official WHO report^b^	Africa and Asia (60s to 80s); Americas, Western Pacific	*Aedes* spp.
Crimean-Congo haemorrhagic fever	Crimean-Congo virus: *Nairovirus* (Bunyaviridae)	Regional outbreaks	Africa, the Balkans, the Middle East, Asia	*Hyalomma* spp.
Dengue	Dengue virus, serotypes DEN 1–4: *Flavivirus* (Flaviviridae)	3.2 million (Americas, South-East Asia and Western Pacific, 2015)	Africa, the Americas, Eastern Mediterranean, South-East Asia, the Western Pacific	*Aedes aegypti* and *Aedes albopictus* (secondary vector)
Japanese encephalitis	Japanese encephalitis virus: *Flavivirus* (Flaviviridae)	68,000 (Asia, estimated per year)	South-East Asia and Western Pacific regions	*Culex* spp.
Rift Valley fever	Rift Valley virus: *Phlebovirus* (Bunyaviridae)	Regional outbreaks	Africa, Arabian Peninsula	*Aedes* spp.
Tick-borne encephalitis	Tick-borne encephalitis virus: *Flavivirus* (Flaviviridae)	10,000–12,000 (estimated per year)	Europe, northern China, Mongolia, the Russian Federation	Ixodidae
West Nile fever	West Nile virus: *Flavivirus* (Flaviviridae)	Regional outbreaks	Africa, Europe, the Middle East, North America and West Asia	*Culex* spp.
Yellow fever	Yellow fever virus: *Flavivirus* (Flaviviridae)	200,000 (estimated per year)	Africa, Central and South America	*Aedes* and *Haemagogus*
Lyme disease	*Borrelia burgdorferi* (Spirochaetaceae)	25,359 (USA, 2014)^c^	Areas of Asia, north-western, central and eastern Europe, USA	Ixodidae
Plague	*Yersinia pestis* (Enterobacteriaceae)	783 (2013)	Asia and South America (until 90s); Africa	*Xenopsylla cheopis*
Rickettsiosis	Species of the genera: *Rickettsia*, *Orientia*, *Ehrlichia*, *Neorickettsia*, *Neoehrlichia* and *Anaplasma*	Millions of cases annually^c^	Americas, Europe, Asia, Africa	Ticks, lice and fleas
Tularaemia	*Francisella tularensis* (Francisellaceae)	Regional outbreaks	North America, eastern Europe, China, Japan, Scandinavia	*Dermacentor* spp.*, Chrysops* spp.*, Amblyomma americanum*
American trypanosomiasis (Chagas disease)	*Trypanosoma cruzi* (Trypanosomatidae)	6 to 7 million	Central and South America	Triatominae
African trypanosomiasis (sleeping sickness)	*Trypanosoma brucei* (Trypanosomatidae)	3,796 (2014)	sub-Saharan Africa	*Glossina* spp.
Leishmaniasis	*Leishmania* spp*.* (Trypanosomatidae)	900,000–1.3 million (estimated per year)	Americas, North Africa-Eurasia, East Africa, South-East Asia, Mediterranean basin	Plebotomine sand flies
Malaria	*Plasmodium* spp*.* (Plasmodiidae)	214 million (estimated, 2015)	sub-Saharan Africa, Asia, Latin America, the Middle East	*Anopheles* spp.
Lymphatic filariasis	*Wuchereria bancrofti* (Onchocercidae)	120 million (2000)	Angola, Cameroon, Côte d’Ivoire, Democratic Republic of the Congo, India, Indonesia, Mozambique, Myanmar, Nigeria, the United Republic of Tanzania	*Culex* spp.
Onchocerciasis	*Onchocerca volvulus* (Onchocercidae)	25 million^c^	sub-Saharan Africa, Yemen, Brazil, Venezuela	*Simulium* spp.
Babesiosis	*Babesia* spp*.* (Babesiidae)	1,762 (USA, 2013)^c^	EUA	Ixodidae

Data from World Health Organization (WHO) web page available in <http://www.who.int/en/>. Accessed on September 15, 2016

^a^Data from Brazilian Health Ministry

^b^Recent outbreak in South and Central America but no official count of the number of people infected was reported by WHO

^c^Data from Centers for Disease Control and Prevention (CDC) web page available in <http://www.cdc.gov>. Accessed on September 15, 2016

Ecological factors are associated with vector dispersion to urban areas [3]. Ticks, triatomine bugs, mosquitoes, sand flies, tsetse and black flies are the main haematophagous arthropod vectors [2], which present different feeding habits. In ticks and triatomines, this habit is seen in both female and male, and in all stages of development. Changing from one stage to the next requires at least one blood meal. On the other hand, only females of mosquitoes and sand flies require a blood meal to fulfil their need to complete the oogenesis process [4].

Vascular damage caused by the haematophagous bite during the repast triggers physiological defence responses in the host that are mainly determined by three important events: haemostasis, immunity and inflammation. To accomplish a continued blood flow, a saliva array of pharmacologically active biomolecules, as antihaemostatic, anti-inflammatory and immunomodulatory compounds, is injected into the bite site [5–9]. Within this context, different pathogens can be transmitted by vector saliva [10, 11]. Depending on each feeding habit, after achieving the necessary fluidity, the haematophagous can consume a large amount of blood in a single meal, and proceed to digestion [4]. Various proteases are involved in the blood meal digestion as a means to obtain the necessary energy for vital biological processes, guaranteeing the haematophagous arthropods’ survival, biological development and reproduction [11].

Proteases are enzymes that hydrolyse (a) peptide bond(s) in amino acid residue sequences; if such catalysis occurs in internal peptide bonds of a protein, they are called endopeptidases. However, when cleavage of a peptide bond takes place at the N- or C-terminal of a polypeptide chain, those enzymes are named exopeptidases. Protease classification involves the clustering of related sequences into families. Currently, there are seven main different families of proteases: aspartic, cysteine, glutamic, metallo, serine, threonine peptidase and asparagine lyase, all grouped according to the molecular composition of their active sites [12]. The clans represent one or more families that have evolutionary relationships evidenced by their tertiary structures or, when no tertiary structures are available, by the order of amino acid residues in the catalytic site and/or by common sequences around it [12]. Each clan is identified by two letters where the first represents the catalytic type of the families. There are three additional letters to assign a clan: P, for peptidases of mixed catalytic type; U, for peptidases of unknown catalytic type; and I, for inhibitors that are proteins. A clan identifier example is PA, which contains both serine PA(S) and cysteine peptidases PA(C). Regarding the family identification, it contains a letter representing the peptidase catalytic type together with a unique number [12]. For instance, S1 is the family of trypsin and chymotrypsin that also belongs to the PA(S) clan. Another clan example is CA, which contains several families of cysteine peptidases with structures like that of papain [12]. In this clan, C1 is the family of cathepsin B and L, peptidases that may act in the digestive vacuoles of protozoa and/or in the lysosomal system of eukaryotic cells [13].

Proteolytic enzymes may be synthesized as zymogens (inactive precursors) or as inactive forms bound to natural inhibitors to prevent unwanted protein degradation as well as to facilitate spatial and temporal organization of proteolytic activity [14]. Zymogen conversion to the active enzyme occurs by limited proteolysis and removal of an activation segment from its tertiary structure within an appropriate subcellular compartment or at the extracellular environment. Proteolysis of the activation segment may be performed by another peptidase or by autocatalysis, requiring, for instance, a drop in pH [14]. In this review, we highlight the functions of haematophagous arthropod proteases in blood-dependent biological processes, with an emphasis on their roles in vector biology.

## The role of arthropod vector proteases in blood dependent processes

### Haematophagy

Haematophagous arthropod vectors tend to take large blood meals, reducing the number of host visits and ensuring a supply of nutrients for a long period [4]. The blood-feeding habit can both occur from haemorrhagic pools that accumulate in the tissues following skin lacerations (pool feeders, as sand flies and ticks) or directly from a cannulated venule or arteriole (vessel feeders, as triatomines and mosquitoes) [15].

Haemostasis aims to restore vascular architecture and prevent blood loss leading to vasoconstriction, platelet aggregation and clotting [16, 17]. These would disrupt feeding and bleeding. Haematophagous saliva is injected at the bite site continuously during probing and ingestion phases to recognize and neutralize/modulate molecules involved in critical haemostatic pathways [17–20]. Among anti-haemostatic mechanisms, there is a variety of salivary natural protease inhibitors, pointing to the diverse cocktail arthropods produce against host proteases [21–24].

Advances in transcriptomic approaches have made it possible to analyse in a deeper insight the biochemical complexity of the saliva from many haematophagous arthropods, unravelling coding sequences for salivary gland proteases [25–36]. However, these sequences are not a guarantee of salivary protein expression, and few have been characterized so far [37]. From our experience, the saliva of triatomine bugs displays low proteolytic activities, tested by in-gel zymography or saliva direct incubation with fluorogenic substrates (unsubmitted).

### Digestion

Proteins represent about 95% of the blood [4], from which albumin and haemoglobin (Hb) comprise over 80% of the total protein content [38]. Consequently, the haematophagous arthropods require proteases as the main enzymes in the midgut to process blood meal digestion [4, 38].

The blood meal is placed in the gut lumen, and it is usually separated from the epithelium by an extracellular semipermeable layer, known in some species as peritrophic matrix [4]. In insects, the architecture of the gut is usually a simple tube constituted of one layer of epithelium resting on a continuous basal lamina. There are functional variable sections in the gut among the different insect orders, but generally a uniform pattern can be observed. The anterior segment receives the blood meal and displays specializations consistent with the abilities to post-feeding distension, ion and water regulation to dehydration of blood, and carbohydrate digestion; while the posterior segment is often responsible for the synthesis and secretion of digestive proteases to digest the meal [39, 40]. The tick midgut consists of a central stomach that acts as a storage organ. Histologically, the lumen is surrounded by a thin epithelial layer and a thin outer layer of muscle fibers [11, 41].

**Fig. 1:**
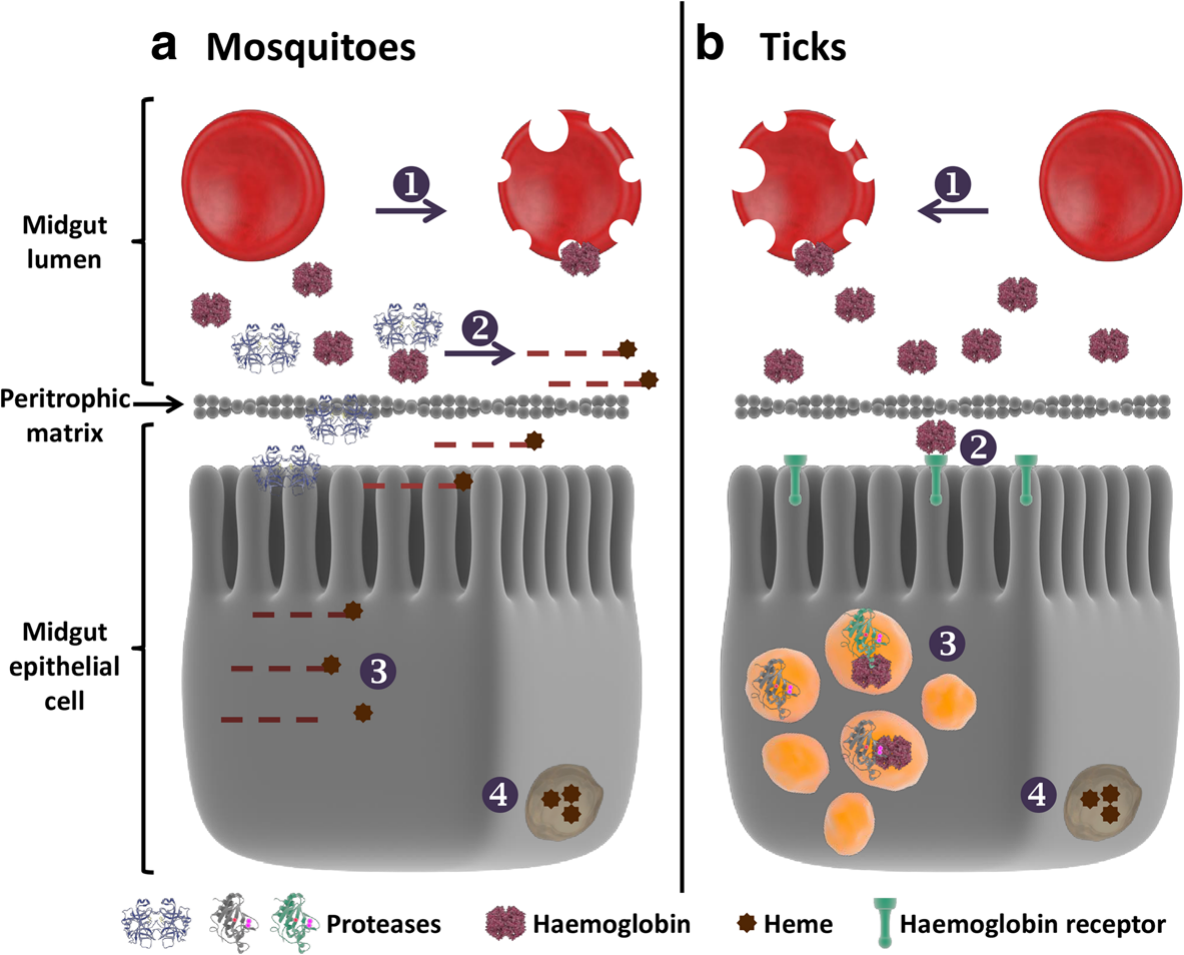
Haemoglobin digestion in mosquitoes and ticks. **a** Host erythrocytes undergo lysis and release haemoglobin (Hb) and other proteins in the lumen of the midgut (1). In mosquitoes, proteases are secreted in the gut lumen for initial Hb extracellular digestion (2), generating peptides that will be further internalized and hydrolyzed in the epithelial cells (3). **b** In ticks, Hb is internalized by receptor-mediated endocytosis (2) and directed to large endosomal vesicles that fuse with lysosomes containing cysteine and aspartic proteases where it is degraded (3). Upon degradation of Hb, free heme must be detoxified (4)

Haematophagous arthropod vectors can be divided in two groups based on the different strategies to process the blood digestion [38]. In insects, midgut cells synthesize and secret digestive proteases in the lumen, typically *via* secretory vesicles or other small secretory structures placed near the base of the microvilli, where extracellular digestion occurs generating peptides, which are then absorbed by the epithelial cells [4, 39] In haematophagous insects the proteolytic network involved in midgut protein digestion is composed by serine proteases, mainly trypsins, with chymotrypsins and carboxypeptidases playing a supplementary role (Fig. 1). In this group, the triatomines are an exception as they use cathepsin and aspartic proteases [4].

In ticks, the digestion process occurs intracellularly through heterophagy by midgut cells [41]. Albumin is taken non-specifically by endocytosis into small acidic vesicles, while the endocytosis of Hb by digestive cells would be mediated by specific receptors and addressed to large digestive vesicles [42, 43]. Although the internalization of albumin and Hb by digestive cells occur by distinct routes, the proteolytic system that controls the albuminolytic and haemoglobinolytic pathways is the same [38]. A multi-enzyme model for Hb degradation was proposed in *Ixodes ricinus*. Inside the acidic digestive vesicle, the degradation pathway is initiated by cysteine and aspartic endopeptidases (cathepsin L, legumain and cathepsin D), generating large peptides fragments (8–11 kDa), followed by the action of cathepsins B and C exopeptidases, generating smaller peptides (2–7 kDa). Finally, serine carboxypeptidase (SCP) and leucine aminopeptidase (LAP) might participate in the liberation of dipeptides and free amino acids. It has been suggested that the final stages of Hb degradation take place both in and outside of the digestive vesicles, in the cytosol. The heme moiety released forms aggregates that are accumulated in the hemosomes. The Hb specific receptor probably evolved as an adaptation to avoid the toxicity of the heme (Fig. 1) [11, 44, 45].

### Yolk formation

A blood meal provides the necessary resources for haematophagous arthropods to produce their eggs [4]. The yolk precursor protein vitellogenin (Vg) is, in arthropods, synthesized in the fat body and then secreted into the haemolymph. After being uptaken by oocyte coated vesicles, the Vg suffers dissociation and a crystallization process occurs in the endosome compartment, forming the yolk body. Vitellogenin proteolysis generates vitellin (Vt) in lysosome-like organelles. The final mature yolk body containing the crystalline Vt form provides the energy to support embryo development, together with lipids and sugars [46].

The accumulation of yolk proteins is regulated by the developmental hormones juvenile hormone (JH) and 20-hydroxyecdysona (20-HE), both found in low levels in young females. Once adults undergo eclosion, the level of JH rises and the fat body becomes responsive to signals that induce vitellogenesis [47]. The roles of JH also include the growth of terminal follicles and the development of oocyte competence for protein internalization [46].

**Fig. 2:**
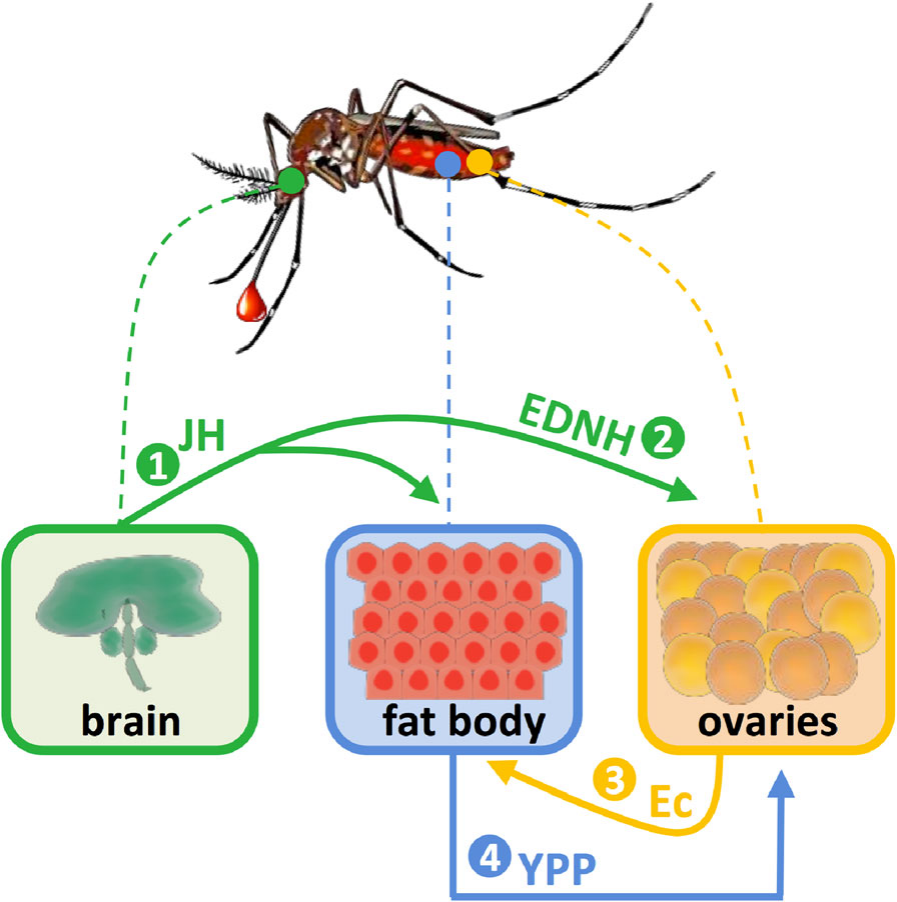
Hormonal control of yolk formation. There are three invertebrate hormones that play major roles in yolk formation. Young females have a high level of juvenile hormone (JH), which is produced by *corpora allata* located in the arthropod brain and acts on fat body and ovaries (1). Upon a blood meal, the JH level drops in haemolymph, and egg development neurosecretory hormone (EDNH) (2) level increases to stimulate the release of ecdysone (Ec) (3) by ovaries that is converted in 20-hydroxyecdysona (20-HE) in the fat body. Together with 20-HE, the nutrients consumed during the blood meal stimulate the expression and secretion, by fat body cells, of yolk precursor proteins (YPP) (4) that are essential in vitellogenesis

Upon a blood meal, the JH level drops in haemolymph, while that of the egg development neurosecretory hormone (EDNH) increases to stimulate the release of ecdysone by ovaries. The latter is a steroidal prohormone that is converted in 20-HE, the main regulator of vitellogenesis in the fat body. This hormone stimulates the expression of Vg, which is secreted in the haemolymph and endocytosed by oocytes [47]. Besides the 20-HE, the nutrients consumed during a blood meal could also be a signal for vitellogenesis regulation by the fat body [48]. The hormonal regulated yolk formation steps are summarized in Fig. 2.

### Immunity

All arthropods need to defend themselves against infectious pathogens. Their innate immune response has physical barriers that include the cuticle, gut, trachea, chemical barriers, and defender cells that allow well-developed humoral and cellular responses [49]. The humoral responses are accomplished by antimicrobial peptides, such as defensins, secreted by fat body, hemocytes and epithelial layer of the gut [49, 50].

**Fig. 3:**
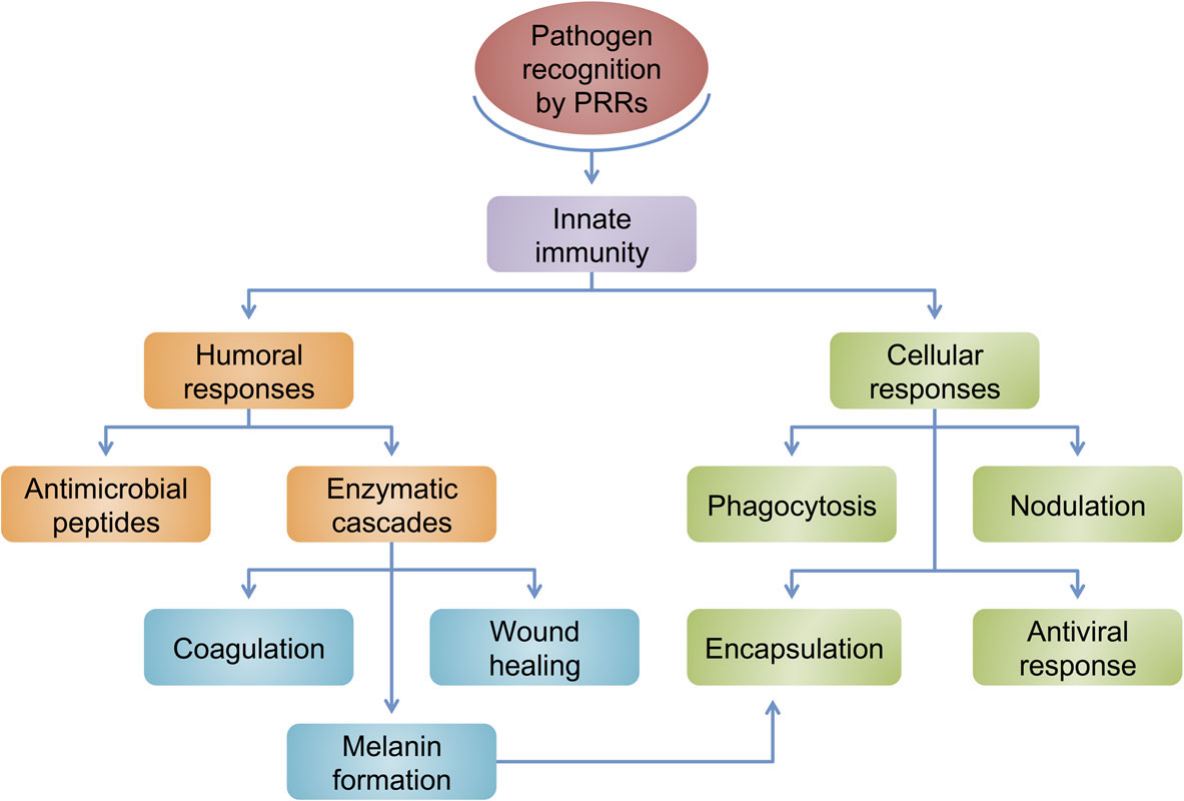
Overview of the arthropod innate immune system

Pathogen recognition occurs *via* soluble or transmembrane pattern recognition receptors (PRRs) that respond to pathogen-associated molecular patterns (PAMPs), resulting in antimicrobial peptides synthesis, enzymatic cascades that can induce coagulation of haemolymph, wound healing and melanin formation. In addition, it may also activate phagocytosis, encapsulation, nodulation and antiviral response. Our current view of the arthropods immune system is represented in Fig. 3. Three major arthropod signalling immune pathways involved in the humoral and cellular responses have been described: the Toll, the immunodeficiency (IMD), and the JAK-STAT [51]. Innate immune response is triggered upon activation of Toll and IMD pathways, inducing antimicrobial peptide gene expression [52]. The JAK-STAT pathway also exerts its activity against such pathogens as viruses [53], bacteria [54–56], and *Plasmodium* [55, 57].

Among the enzymatic cascades, the prophenoloxidase (proPO) one leads to melanisation of pathogens and damaged tissues, one of the major innate defence systems in invertebrates. Tiny amounts of PAMPs recognized by the PRRs ensure the activation of zymogenic proPO into active phenoloxidase (PO) by a cascade of serine proteases. PO oxidizes tyrosine to dihydroxyphenilalanine and then to quinones, which are precursors of melanin and other toxic and reactive compounds. This process is controlled by specific protease inhibitors and by active PO in a complex manner aiming to avoid superfluous activation and production of cytotoxic compounds [51, 58, 59].

Finally, the presence of hypervariable PRRs in arthropods [60–62] with the ability to differently bind and recognize a range of microorganisms, microbial products, and multicellular parasites has shed some light on the possible existence of memory and specificity in arthropod immunity [62–64].

## Proteases from haematophagous arthropod vectors

### In ticks

The digestive proteolytic network from *I. ricinus* proposed by biochemical and genetic analyses indicated a combination of four cysteine peptidase activities, cathepsins B (*Ir*CB), C (*Ir*CC), L (*Ir*CL) and legumain (*Ir*AE), and an aspartic peptidase activity, cathepsin D (*Ir*CD) that operate together in haemoglobinolysis [65]. An insight into the gene transcription revealed that an increase in total haemoglobinolysis matches with the activity profiles of *Ir*CC, *Ir*AE, *Ir*CD and *Ir*CB, being the last the most abundant protease of the pathway [66]. The endolysosomal localisation of *Ir*CL1 was confirmed by immunolocalization [67]. The legumain *Ir*AE is expressed only in the gut tissue and is localized within the peritrophic matrix, beyond in the digestive vesicles of gut cells. *Ir*AE hydrolyzed Hb to a predominant peptide of 4 kDa [68].

Cathepsin L-like cysteine proteases have been reported in *Haemaphysalis longicornis* [69], HlCPL-A is up-regulated during the repast and cleaves bovine Hb in a dose-dependent manner at pH 5.5 [69]. Two other cathepsin L-like genes, HLCG-A and HLCG-B may also have important functions in the digestion of host Hb [70]. These cathepsin L-like cysteine activities are also present in *Rhipicephalus* (*Boophilus*) *microplus* tick crude midgut extracts [71–73], larvae [74, 75], and eggs [76]. The enzymes mediating these activities are named *Boophilus microplus* cathepsin L-like (BmCL1), *R. microplus* larval cysteine endopeptidase (RmLCE), and vittelin degrading cysteine endopeptidase (VTDCE), respectively. RmLCE is possibly the native form of the recombinant BmCL1 [74]. VTDCE is present in fat body, gut, salivary glands, ovary extracts, and haemolymph from partially or fully engorged females, suggesting it could have an extra ovarian origin, to be later internalized by oocytes [76]. Coexistence has been proposed between VTDCE and Vg/Vt with no polypeptide cleavage during vitellogenesis [77]. Although VTDCE has been classified as a cathepsin L-like cysteine [76], a very low similarity was found between its deduced amino acid sequence (AFK78425.1) and any other cysteine endopeptidase. On the other hand, phylogenetic sequence analysis revealed that VTDCE is similar to some tick antimicrobial peptides [78]. Moreover, the presence of VTDCE significantly inhibits *Staphylococcus epidermidis* growth after a period of 24 h. This is the first arthropod protease to be reported as an antimicrobial that is not correlated with its peptidase activity [78]. Finally, VTDCE, BmCL1 and RmLCE hydrolyse Hb and vitellin at acidic pH [73, 74, 76], and thus may have a fundamental role during tick development.

Taking into consideration the works mentioned above had been published before the *R.* (*B.*) *microplus* genome sequencing [79], we decided to carry out a deeper investigation to differentiate the sequence annotations and features of those three proteases. After a search into *R.* (*B.*) *microplus* genome database (GenBank: HM748961), ten different protein-coding genes for cathepsin L were identified, including BmCL1 (AAF61565.1); nevertheless, none of them codes for VTDCE. A comparative pair wise amino acid sequence alignment demonstrates a homology of, at least, 97% among the sequences (Table 2), that together with the fully identified active site residues (Additional file 1) may indicate *R.* (*B.*) *microplus* presents ten active cathepsin L isoforms. It is not possible to conclude that BmCL1 and RmLCE are the same isoform. However, a stage specific expression pattern may exist to guarantee the success of cathepsin L blood dependent processes in this tick.

**Table 2:** Percentage of sequence identity between predicted Cathepsin L from *Rhipicephalus* (*Boophilus*) *microplus* after pairwise alignment performed with EMBOSS Needle

Accession number	AFQ 98389.1	AFQ 98385.1	AFQ 98392.1	AFQ 98386.1	AGK 88363.1	AFQ 98393.1	AFQ 98390.1	AFQ 98387.1	AAF 61565.1 (BmCL1)	AFQ 98388.1
AFQ98389.1	100	99.4	98.5	98.2	98.2	98.2	98.2	98.5	98.2	98.2
AFQ98385.1		100	99.1	98.8	98.8	98.8	98.8	99.1	98.8	98.2
AFQ98392.1			100	98.5	98.5	99.7	99.7	98.8	99.1	97.3
AFQ98386.1				100	98.8	98.2	98.8	98.5	98.8	97.6
AGK88363.1					100	98.2	98.8	98.5	98.8	97.6
AFQ98393.1						100	99.4	98.5	98.8	97.0
AFQ98390.1							100	99.1	99.4	97.6
AFQ98387.1								100	99.1	97.9
AAF61565.1 (BmCL1)									100	97.6
AFQ98388.1										100

Tsuji et al. [80] reported the molecular characterization of a cathepsin B-like named longipain from the midgut epithelium of *H. longicornis* tick. It is specifically localized in the lysosomes and secreted into the intestinal lumen, following blood-feeding. Enzymatic assays with natural substrates indicate that longipain cleaves spectrin, an important component of erythrocyte membranes, but not Hb. Endogenous RNAi knockdown experiment suggests longipain activity in ticks is involved in feeding capacity and protection against parasites [80]. It is worth pointing out that this toxic effect may be direct and/or by means of the degradation of ingested proteins and peptides.

Legumains have been identified in the gut of *H. longicornis*, *H. longicornis* legumain 1 (HlLgm1) and *H. longicornis* legumain 2 (HlLgm2), by their ability to cleave Z-Ala-Ala-Asn-AMC at neutral pH [81, 82]. Differently, the optimal pH activity of *Ir*AE legumain from *I. ricinus* is acidic [68]. HlLgm1 and HlLgm2 localize in the midgut epithelium and are upregulated during the blood-feeding process. However, HlLgm2 is expressed at a lower level than HlLgm1 during digestion and there is no expression of HlLgm2 above 96 h of feeding. The expression of HlLgm1 continues until full engorgement [82]. Moreover, the cleavage of bovine Hb by these legumains corroborates their role in the digestion of blood proteins [83]. Silencing of both genes by RNAi has revealed an extended feeding period, survival decrease, weight loss, delayed oviposition and reduced number of normal eggs. In addition, the epithelium of the gut shows, upon this condition, damage and disruption of normal cellular remodelling during feeding, resulting in luminal narrowing in silenced individuals [83].

The results of some very well designed experiments indicate that three cathepsin D isoforms (*Ir*CD 1–3) play central and distinct roles in the physiology and development of *I. ricinus. Ir*CD1 is associated with the gut of partially engorged female ticks and is induced by feeding. This protease plays a haemoglobinolytic role in the digestive vesicles supported by immunolocalization and RNAi knockdown. *Ir*CD2 isoform is expressed both in gut and salivary glands and its expression peak is observed in fully fed females. *Ir*CD3 isoform is expressed in ovaries, and therefore is not related to haemoglobinolysis [84]. It has been proposed *Ir*CD1 would act together with *Ir*AE, while *Ir*CD2 could be secreted into the gut lumen to generate haemoglobin-derived antimicrobial peptides to preserve the blood meal. Finally, *Ir*CD3 isoform would play a role in yolk protein degradation [85].

Other aspartic proteases have been shown to be also involved in yolk degradation [11]. Eggs of *R.* (*B.*) *microplus* express two aspartic proteases able to degrade Vt during embryogenesis: boophilus yolk cathepsin (BYC) and tick heme-binding protease (THAP). The activity of THAP seems to be regulated by heme molecule, and BYC also cleaves Hb [86–88]. Interestingly, a cathepsin D from this tick midgut (BmAP) may be responsible for the generation of antimicrobial peptides, suggesting that proteases play roles in immune response against parasite invasion [85, 89]. At last, a *H. longicornis* cathepsin D (longepsin) is highly expressed in the midgut after a blood meal and hydrolyses Hb, besides being expressed in the salivary glands [90].

Some SPs have also been described in ticks. A multi-domain SP from *I. ricinus* named *Ir*FC triggers coagulation of haemolymph in response to bacterial lipopolysaccharides, as its homolog in horseshoe crab. RT-PCR analysis has revealed that the *Ir*FC mRNA is expressed in all life stages, and in adults it is present mainly in hemocytes as observed by indirect immunofluorescence microscopy, suggesting this enzyme has a function in tick immunity [91].

HLSG-1 and HLSG-2 SPs of the hard tick *H. longicornis*, which carries and transmits various pathogens [92], are blood meal-induced and expressed in the midgut, salivary glands as well as in other organs [93]. Another *H. longicornis* SP named HlSP is expressed during development and is localized in the adult tick midgut. This protease contains the domains CUB (complement C1r/C1s, Uegf, Bmp1) and LDL (low-density lipoprotein receptor class A domains), important at mediating extracellular protein–protein interactions [94–96]. Lower levels of HlSP upon RNAi correlates well with the diminished capacity of ticks to degrade host erythrocytes, suggesting this enzyme is involved in haemolysis. Moreover, the recombinant protein rHlSP also shows haemolytic activity in vitro in a dose-dependent manner [95]. Two other SPs studied, HlSP2 and HlSP3, are also localized in the midgut epithelial cells and lumen of adult ticks [97]. Silencing of these three SP genes together have resulted in body weight reduction, indicating they may form a proteolytic network for host Hb digestion in the midgut of ticks [97]. Finally, a carboxypeptidase-like SP*,* HlSCP1, is found in the vacuoles of midgut endothelial cells of *H. longicornis*, and its upregulation is observed after a blood meal. Of interest, this protease is also able to cleave Hb [98].

Curiously, *I. scapularis* degradome (the full repertoire of proteases encoded by the genome) is mainly represented by metalloproteases (~40%) [99]. These are organized in 23 families, but functions in tick physiology are unknown for many of them. M12 family contains 14 enzymes that are believed to be involved in the regulation of blood-feeding. For instance, recombinant M12 AAP22067 mediates gelatinase and fibrinogenolytic activities [19], which are essential to maintain host blood in a fluid state during tick feeding. Metalloproteases make part of the midgut transcriptome from the hard tick *Dermacentor variabilis* [100], but their functions are unknown.

From the hard tick *H. longicornis*, an aminopeptidase member of the M17 family, HlLAP, is upregulated by blood meal during initial feeding period and acts in the liberation of free amino acids in the cytosol of midgut epithelial cells [101]. In the sialotranscriptome of *Haemaphysalis flava*, metalloprotease genes supposed to be involved in modulating host haemostasis are over expressed in semi-engorged ticks, probably to maintain blood flow [102]. In *R. microplus,* RNAi silencing of metalloproteases affects average egg weight and oviposition rate [103]. In addition, three metalloprotease sequences from *Amblyoma americanum* show identity to annotated tick metalloproteases, and another shows identity to *I. scapularis* endothelin-converting enzyme (ECE) [104]. Endothelins are a family of potent vasoconstrictive peptides [105]. Thus, the role of ECE in haematophagous arthropod saliva might be the hydrolysis of endothelins to impair vasoconstriction.

Finally, tick salivary glands also express metalloproteases. From *I. ricinus*, two cDNAs coding homologous putative metalloproteases (Metis 1 and Metis 2) are expressed in salivary glands during feeding most likely to stimulate fibrinolysis. Indeed, knock-down by RNAi of Metis 1 and Metis 2 impairs blood meal completion [106]. The presence of specific antibodies against HLMP1, a recombinant tick reprolysin metalloprotease, results in lower feeding efficiency of *H. longicornis* in rabbits [107]. Protein sequences of the reproplysin family of metalloproteases from *Ixodes persulcatus* (Ip-MPs), *Rhipicephalus sanguineus* (Rs-MPs) and *R. microplus* (BrRm-MPs) have been found in the salivary glands of partially and fully fed female ticks, and may be required during tick feeding to manipulate host defences and support tick haematophagy [108].

**Fig. 4:**
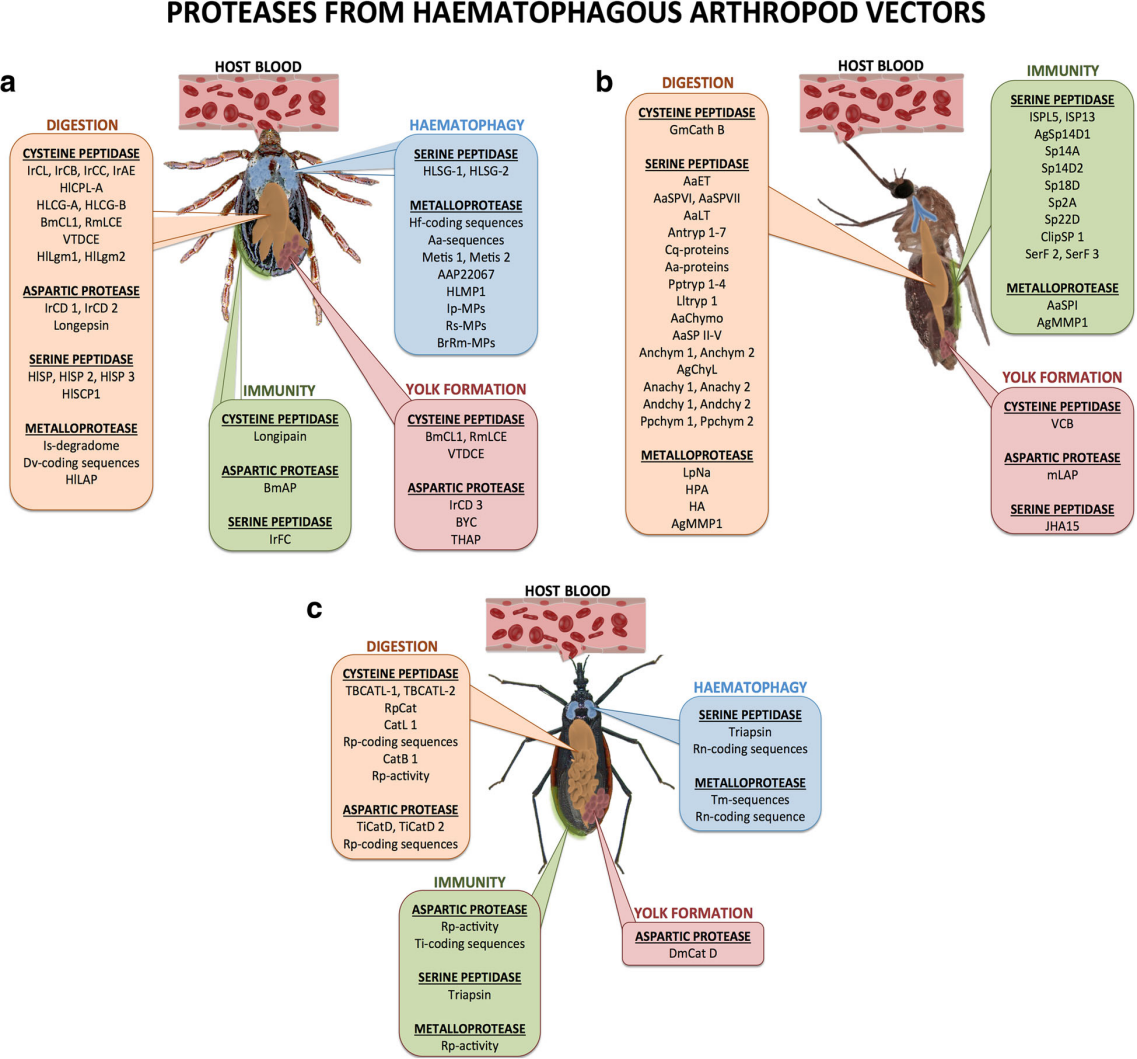
Overview of proteases from haematophagous arthropods. The columns show proteases reported in ticks, arthropods from the class Arachnida (**a**) and also in the orders of the class Insecta: Diptera (Culicidae) (**b**) and Hemiptera (Reduviidae: Triatominae) (**c**). The colours used group the proteases according to their biological function as follows: *orange*, digestion; *blue*, haematophagy; *green*, immunity; and *red*, yolk formation

In summary, cysteine-, serine-, aspartic- and metallo-proteases have been described in ticks (Table 3 and Fig. 4). Notably cysteine and aspartic proteases are known for their role in tick digestion; however, as evidenced, they are distributed in different tissues where they have variable biological functions. Serine proteases are related to digestion and immunity, and metalloproteases have been described in the salivary glands and may act mainly on the vector-host interface to prevent haemostasis. Tick proteases play wide biological roles and their expressions and activities undergo tissue specific regulation.

**Table 3:** Proteases from ticks

Protease	Species	Synthetic/Natural substrate	Inhibitor	Localization	Role	Reference
Cysteine						
Cathepsin L						
IrCL 1	*Ixodes ricinus*	Z-Phe-Arg-AMC	E-64; Z-Phe-Phe-DMK	Gut	Digestion	[67]
HlCPL-A	*Haemaphysalis longicornis*	Z-Phe-Arg-AMC	Leupeptin; Antipain; E-64	Gut	Digestion	[69]
HLCG-A; HLCG-B	*H. longicornis*	–	–	–	Digestion	[70]
BmCL 1	*Rhipicephalus* (*Boophilus*) *microplus*	H-D-Val-Phe-Lys-pNA; Hb	E-64; Leupeptin; Antipain	Gut	Digestion; yolk formation	[72, 73]
RmLCE	*R.* (*B.*) *microplus*	AbzAla-Ile-Ala-Phe-Phe-Ser-Arg-Gln-EDDnp; N-Cbz-Phe-Arg-AMC; Hb	E-64; Pepstatin A; Papain	Larve; gut	Digestion; yolk formation	[72, 74]
VTDCE	*R.* (*B.*) *microplus*	N-Cbz-Phe-Arg-AMC; Hb	E-64	Salivary glands; gut; haemolymph; fat body; ovary; eggs	Yolk formation; immunity; digestion	[76, 77]
Cathepsin B						
IrCB	*I. ricinus*	–	–	Gut	Digestion	[44, 66]
Longipain	*H. longicornis*	Z-Phe-Arg-AMC	E-64	Gut	Immunity	[80]
Cathepsin C						
IrCC	*I. ricinus*	Hb	–	Salivary glands; gut; ovary	Digestion	[44, 66]
Legumain						
IrAE	*I. ricinus*	Z-Ala-Ala-Asn-AMC	Azapeptidil	Gut	Digestion	[68]
H1Lgm 1; H1Lgm 2	*H. longicornis*	Z-Ala-Ala-Asn-AMC	Iodoacetamide; N- ethylmaleimide	Gut	Haematophagy; digestion; yolk formation	[81–83]
Aspartic						
Cathepsin D						
IrCD1	*I. ricinus*	Hb	Pepstatin	Gut female	Digestion	[85]
IrCD2	*I. ricinus*	–	–	Gut; salivary glands	Digestion	[85]
IrCD3	*I. ricinus*	–	–	Ovaries	Yolk formation	[85]
BYC	*R.* (*B.*) *microplus*	Hb; Vitellin	–	Eggs	Yolk formation	[86, 87]
THAP	*R.* (*B.*) *microplus*	Abz-AIAFFSRQ-EDDnp	Pepstatin	Eggs	Yolk formation	[88]
BmAP	*R.* (*B.*) *microplus*	–	–	Gut	Immunity	[89]
Longepsin	*H. longicornis*	FITC-casein	Pepstatin A	Salivary glands; gut	Digestion	[90]
Serine						
Trypsin-like						
IrFC	*I. ricinus*	–	–	Hemocytes	Immunity	[91]
HLSG1; HLSG2	*H. longicornis*	Bz-I-Arg-pNa; BSA		Salivary glands; gut	Haematophagy; digestion	[93]
HlSP; HlSP 2; HlSP 3	*H. longicornis*	Bz-I-Arg-pNa; BSA	–	Gut	Digestion	[94, 95, 97]
Carboxypeptidase-like						
HlSCP1	*H. longicornis*	Z-Phe-Leu; Pyr-Phe-Leu-pNA	PMSF	Gut	Digestion	[98]
Metallo						
Is-degradome	*Ixodes scapularis*	–	–	–	Digestion	[99]
Dv-coding sequences	*Dermacentor variabilis*	–	–	Gut	Digestion	[100]
Hf-coding sequences	*Haemaphysalis flava*	–	–	Salivary glands	Haematophagy	[102]
Aa-sequences	*Amblyomma americanum*	–	–	Salivary glands	Haematophagy	[104]
Metis 1; Metis 2	*I. ricinus*	–	–	Salivary glands	Haematophagy	[106]
M12						
AAP22067	*I. scapularis*	Gelatin	–	Salivary glands	Haematophagy	[19]
M17						
HlLAP	*H. longicornis*	–	–	Gut	Digestion	[101]
Reproplysin						
HLMP1	*H. longicornis*	–	–	Salivary glands	Haematophagy	[107]
Ip-MPs	*Ixodes persulcatus*	–	–	Salivary glands	Haematophagy	[108]
Rs-MPs	*Rhipicephalus sanguineus*	–	–	Salivary glands	Haematophagy	[108]
BrRm-MPs	*R.* (*B*.) *microplus*	–	–	Salivary glands	Haematophagy	[108]

### In triatomines

There are a few reports on triatomine protease activities. In triatomine bugs, two cathepsin L-like proteases of *Triatoma brasiliensis*, TBCATL-1 and TBCATL-2 [109]; one of *R. prolixus,* RpCat [110], and another of *T. infestans* (CatL1) [111] have been characterized in the midgut of these species. TBCATL-1 and TBCATL-2 proteolytic activities have been detected in the posterior midgut by zymogram assay. Cathepsin B (CatB1) is present in gut extracts of *T. infestans*. CatL1 and CatB1 activities decrease during the first two days after feeding but increase to a maximum value at five and 10 days post feeding. A strong acidic peptidase activity found in the gut extract of *T. infestans* is possibly mediated by a cathepsin B. Although the molecular features and functional properties of the protein are unknown, the enzymatic activity is efficiently inhibited by CA-074, a specific cathepsin-B inhibitor [111]. The cathepsin B-like activity, which is present in the midgut of *R. prolixus*, is increased following a blood meal [112]. Indeed, trace amounts of cathepsin B are detected in the lysosome of *R. prolixus* midgut cells before feeding, but after blood sucking, cathepsin B localizes in a granular precipitate associated with this organelle, and may be released in the gut lumen [113].

A cathepsin D aspartic-like protease activity is detectable in the blood-sucking triatomine *R. prolixus* [114]. It has been suggested that *Trypanosoma cruzi* colonization of *R. prolixus* may modulate the expression of cathepsin D in the invertebrate since its activity is much higher on days 1–3 after infection [114]. A similar result has been found in *T. infestans* [115], another vector of *T. cruzi*. Yet, *T. infestans* midgut TiCatD is strongly induced after feeding whereas TiCatD2 is upregulated only 10 to 20 days after meal, suggesting that the former might play a role in processes related to early digestion [116]. The midgut transcriptome of *R. prolixus* shows that transcripts from digestive enzymes are significantly well expressed, with a predominance of cysteine and aspartic proteases [117].

More recently, triatomine cathepsin D has also been proposed to be involved in vitellogenesis. *Dipetalogaster maxima* cathepsin D (DmCatD) is expressed in the fat body and ovarian tissues during the reproductive cycle. As for other peptidases, DmCatD also degrades Vt. Early activation of DmCatD seems to be a relevant physiological mechanism in yolk protein degradation during follicular atresia to either increase female lifetime or sustain younger oocytes until improvement of nutritional conditions [118].

Triapsin is the best serine protease characterized in triatomines. This trypsin-like SP is expressed in the D2 pair of *T. infestans* salivary glands as an inactive precursor and activated during salivation stimulated by biting. Triapsin shows high specificity towards arginine at the P1 site. This protease may be involved in hydrolysis of the superfamily of Proteinase Activated G protein-coupled Receptors (PAR), which regulates growth, development, inflammation, and responses to injury. Triapsin is unlikely to be involved in digestion since this phenomenon in Hemiptera seems to depend exclusively on the action of cysteine and aspartic proteases [18]. However, it is imperative to perform experiments to test the involvement of this peptidase on the physiology of triatomines and other insect vectors of illnesses.

Our group has used next-generation sequencing and mass spectrometry-based protein identification to study the transcriptome and proteome of *R. neglectus* salivary glands (sialome) [25]. The results have revealed abundant transcripts of putative secreted trypsin-like peptidases, although only one SP was detected in the proteome, suggesting physiological conditions may influence secretion [25]. Sequence alignments disclosed the presence of domains present in proteins that act in haemostasis and immunity such as the CUB domain [119] and the cysteine-stabilized structures for molecular recognition (CLIP, LDLa and SUSHI domains). Five SP sequences from *R. neglectus* sialotranscriptome [25] match to SPs sequences from *T. infestans* [28, 120], *T. braziliensis* [29], *P. megistus* [30] and *R. prolixus* [117]. Although physiological roles of SPs are unknown, their presence in the sialotranscriptome of different triatomine species is indicative of the importance of these proteases in haematophagy.

Two metalloproteases are expressed in the haemolymph of *R. prolixus* infected with *Enterobacter cloacae* [121] or *Trypanosoma rangeli* [122]. The source of these proteases is the fat body and their release into the haemolymph upon infection suggests these enzymes may be involved in *R. prolixus* defence mechanisms. In *T. matogrossensis* saliva [123] and *R. prolixus* oddities [117], sequences related to the astacin family of metalloproteases have been reported. In the sialome of *R. neglectus*, one coding sequence related to the zinc-dependent metalloproteases from the astacin-like metalloproteases as well as other two related to the adamalysin/reprolysin family, which includes ADAMTS (A Disintegrin and Metalloproteinase with Thrombospondin motifs), have also been reported. Both are members of the metzincins metalloproteases superfamily [25]. Astacin family members can hydrolyse fibrinogen and fibronectin [124–126], leading to local haemorrhage [127], whereas disintegrins bind to platelets acting as potent inhibitors of platelet aggregation [128–130]. These proteases may have key functions in the maintenance of blood flow at the bite site. In summary, in triatomines vectors, as observed in ticks, protease activities involved in digestion belong to cysteine and aspartic families (Table 4 and Fig. 4), whereas serine- and metallo- proteases seem to participate in some aspects of haematophagy and immunity.

**Table 4:** Proteases from triatomines

Protease	Species	Synthetic/Natural substrate	Inhibitor	Localization	Role	Reference
Cysteine						
Cathepsin L						
TBCATL-1; TBCATL-2	*Triatoma brasiliensis*	Gelatin	E-64; CA-074	Gut	Digestion	[109]
RpCat	*Rhodnius prolixus*	–	–	Gut	Digestion	[110]
CatL1	*Triatoma infestans*	–	–	–	Digestion	[111]
Rp-coding sequences	*R. prolixus*	–	–	Gut	Digestion	[117]
Cathepsin B						
CatB1	*T. infestans*	Z- Phe-Arg-pNA	E-64; CA-074	Gut	Digestion	[111]
Rp-activity	*R. prolixus*	N-Benzoyl-DL-Arg-β-Napthylamide	–	Gut	Digestion	[113]
Aspartic; Cathepsin D						
Rp-activity	*R. prolixus*	Hb	Pepstatin A	Gut	Immunity	[114]
Ti-coding sequences	*T. infestans*	–	–	Gut	Immunity	[115]
TiCatD; TiCatD2	*T. infestans*	MOCAc-GKPILFwFRLK(Dnp)-D-R-NH2	Pepstatin A	Gut	Digestion	[116]
Rp-coding sequences	*R. prolixus*	–	–	Gut	Digestion	[117]
DmCatD	*Dipetalogaster maxima*	–	–	Fat body	Yolk formation	[118]
Serine; Trypsin-like						
Triapsin	*T. infestans*	pNA; H-D-Ile-Pro-Arg-pNA	APMSF; SBTI; Antipain	Salivary glands	Haematophagy	[18]
Rn-coding sequences	*Rhodnius neglectus*	–	–	Salivary glands	Haematophagy	[25]
Metallo						
Rp-activity	*R. prolixus*	Gelatin	1–10 Phenanthroline	Fat body; haemolymph	Immunity	[121, 122]
Astacin						
Tm-sequences	*Triatoma matogrossensis*	–	–	Salivary glands	Haematophagy	[123]
Rp-sequences	*R. prolixus*	–	–	Oddities	–	
Rn-coding sequence	*R. neglectus*	–	–	Salivary glands	Haematophagy	[25]
Reprolysin						
Rn-coding sequence	*R. neglectus*	–	–	Salivary glands	Haematophagy	[25]

### In mosquitoes

Vitellogenic cathepsin B (VCB) from *Aedes aegypti* mosquito is specifically expressed by fat body during vitellogenesis in response to repast, internalized by developing oocytes, and deposited in the yolk bodies for the onset of embryogenesis. VCB hydrolyses Vg at acidic pH, and may be involved in the embryonic degradation of yolk proteins [131].

In female *Ae. aegypti* two different groups of gut trypsins show different levels of expression after a blood meal. The first one is the early trypsin group, whose transcription occurs in the midgut of newly emerged adult before feeding and is under the control of JH [132], being translated at detectable levels after blood-feeding [133, 134]. Early trypsins, secreted either through stretching of the midgut or osmotic effect, would be required for the transcription of the second group, the late trypsins. It is possible that released amino acids are also involved in this process [135, 136]. Expression of late trypsins requires complete synthesis of new mRNAs after feeding, produced in large amounts 8–10 h after a blood meal, suggesting it may have a major role in the digestive process [133]. These two phases would allow the mosquitoes to assess the quality of the meal before committing to the synthesis of late trypsins, since large amount of these proteases in the absence of blood might be harmful for the mosquitoes [135].

Within this context, three trypsins of *Ae. aegypti* [Aedes Early Trypsin (AaET), AaSPVI and AaSPVII] had their enzymatic activities compared among them and with bovine trypsin (BvT). The specific activities of AaET and BvT are comparable, and 5–10 times higher than those of AaSPVI and AaSPVII late trypsins. In addition, AaSPVI is 3–4 times more active on Hb than AaET and AaSPVII [137]. AaSPVI RNAi knockdown, but not that of AaSPVII, triggers a significant decrease in the late phase trypsin-like activity. In contrast, injections of AaSPVI and AaSPVII dsRNAs decrease both degradation of endogenous serum albumin in vivo and egg production. Taken together, these data indicate that AaSPVI and AaSPVII contribute to blood digestion and oocyte maturation [138]. *Ae. aegypti* late trypsin (AaLT), that lacks trypsin-like activity, and AaSPI are classified as collagenase-like SP and might be related to mosquito defence against complement present in the host blood [139].

A gene cluster of gut trypsin-like SPs, Antryp 1–7, has been identified in *Anopheles gambiae* [140–142]. Antryp 1 and 2 present selective proteolytic activity against blood components; Antrypl mediates degradation of both Hb and serum albumin, whereas Antryp2 seems to be mainly active on Hb. While transcription of Antryp 1 and 2 is induced after a blood meal, Antryp 3–7 are constitutively transcribed in females and their levels are down-regulated after blood-feeding. These observations suggest that Antryp 3–7 are involved in initiating the events leading to the expression of other SPs directly associated with digestion [140–142]. Trypsin-like SPs activities have also been observed in *An. aquasalis* females [143]. In the *Aedes* [144] and *Anopheles* [145] these proteases share an acidic isoelectric point, but differ in size.

Regarding *Culex quinquefasciatus*, a proteomic approach associated with zymographic analysis has identified eight trypsin-like proteases in the midgut of females fed on sugar [146]. These enzymes are specific to *C. quinquefasciatus* when compared to the culicids genomes sequenced so far. Moreover, these proteases exhibit singularities at the protein sequence level such as the presence of different amino acids at the autocatalytic motif and substrate binding regions [146].

A female specific *Ae. aegypti* chymotrypsin-like SP gene (JHA15 or AaJA15) is required in the yolk for embryo development and is regulated in a dose-dependent manner by JH [147]. Five other *Ae. aegypti* chymotrypsin-like SP genes (AaChymo, AaSP II-V) have been cloned and sequenced [139]. Northern and Western blots analyses have shown that AaChymo mRNA is abundant in the adult female midgut and its expression is induced after a blood meal [148]. On the other hand, midgut AaSP II–V are equally expressed before and after a blood meal [139]. An explanation for this observation awaits further investigations. In *Ae. albopictus* females two different trypsin-like SPs and one chymotrypsin have been identified by means of two-dimensional electrophoresis of midgut proteins [149].

A study of two midgut *An. gambiae* chymotrypsins (Anchym1 and Anchym2) has revealed the presence of N-terminus preceded by an arginine, indicating zymogen activation by tryptic cleavage. It has been suggested that these chymotrypsins are members of a digestive cascade initiated upon tryptic activation [150]. In the *An. gambiae* midgut the chymotrypsin (AgChyL) is restricted to the adult female stage and contains a Thr residue at the position 182, a feature that could determine its narrow specificity range [151]. In other two species of *Anopheles, An. aquasalis* (Anachy1 and Anachy2) and *An. darlingi* (Andchy1 and Andchy2), two closely related chymotrypsins have also been reported. Anachy1 and Anachy2 mRNAs seem to be detectable only in adult females, approximately 24 h after the blood meal [152].

An intriguing possibility is that SPs levels in haematophagous vectors may be associated with infections. Eight immune related SPs have been described in *An. gambiae:* ISPL5, ISP13 [153, 154], AgSp14D1 [155], Sp14A, Sp14D2, Sp18D, Sp2A [156], and Sp22D [156, 157]. These SPs, except for Sp18D and Sp2A, probably participate in the anti-bacterial and anti-*Plasmodium* defence mechanisms [153–155]. Furthermore, AgSp14D1 catalytic domain has similar sequence identities to kallikreins and coagulation factors, members that are involved in immune and wound responses [155]. Sp14A, Sp14D1, and Sp14D2 present an amino-terminal clip domain, characteristic of secreted proteases that activate prophenoloxidases, regulate melanotic parasite encapsulation and antimicrobial peptide synthesis [155]. Regarding Sp22D mRNA, it is expressed constitutively in three immune related cell types: adult hemocytes, fat body, and midgut epithelial cells. The authors suggest that Sp22D is secreted into the hemolymph where it may interact with pathogen surfaces and initiate an immune response as rapid as pathogen detection [157]. Sp2A and Sp18D functions remain to be characterized, although Sp18D present the clip domain and Sp2A is similar to vertebrate and invertebrate blood coagulation factors [155]. In *An. dirus*, the main vector of malaria in Southeast Asia, the SP cDNAs for ClipSP1, SerF2, and SerF3 have been analyzed upon *P. falciparum* infection. Only SerF3 seems to be upregulated in infected *An. dirus*, and might also play a role in the mosquito immunity [158].

Metalloproteases have also been reported in mosquitoes. Late metalloprotease trypsin, leucineaminopeptidase (LpNa), carboxypeptidase A (HPA) and carboxypeptidase B (HA) of the midgut of *Ae. aegypti* females present enzymatic activities stimulated 20–24 h after a blood or protein, but not free amino acids meal. There is a positive correlation between metalloprotease activity and protein concentration in the meal [159]. AgMMP1, a matrix metalloprotease (MMP) from *An. gambiae,* is expressed as a trans-membrane/membrane protein (MT-MMP1) in epithelial tissues and as a secreted (S-MMP1) isoform in hemocytes. MT-MMP1 transcript levels show a remarkable response to blood meal digestion and to midgut invasion by *Plasmodium* ookinetes [160]. Since tissue invaded by pathogens has often been associated with increased MMP activity, this study suggests MMPs may have an impact in vector competence determination [161, 162].

The mRNA of lysosomal aspartic protease (mLAP) of *Ae. aegypti* females is expressed in fat body during vitellogenesis and is upregulated between 6 and 12 h after blood meal, with high levels at 24 h and then gradually declining. It has been suggested a negative translational regulation of mLAP expression by 20-HE [163].

In summary, the vast majority of mosquito peptidases are serine proteases involved in digestion (Table 5 and Fig. 4), and evidences indicate that they probably participate in other processes, including immunity and development, suggesting they are multitasking enzymes. Interestingly, a member of the metalloprotease class is also involved in digestion. Additionally, although products coding for serine proteases from salivary gland transcriptome have been reported in some mosquito species, the question of whether they have a function in saliva remains to be investigated.

**Table 5:** Proteases from dipterans

Protease	Species	Synthetic/Natural substrate	Inhibitor	Localization	Role	Reference
Cystein						
Cathepsin B						
VCB	*Aedes aegypti*	Z-Arg-Arg-pNA	E-64; Chymostatin	Fat body	Yolk formation	[131]
GmCath B	*Glossina morsitans*	Hb; Serum albumin	–	Gut	Digestion	[170]
Cathepsin D						
mLAP	*Ae. aegypti*	–	–	Fat body	Yolk formation	[163]
Serine; Trypsin-like						
AaET; AaSPVI; AaSPVII	*Ae. aegypti*	BApNA	–	Gut	Digestion; yolk formation	[137, 138]
AaLT	*Ae. aegypti*	–	–	Gut	Digestion; yolk formation	[136, 137]
Antryp-7	*Anopheles gambiae*	–	PMSF	Gut	Digestion	[140–142]
Cq-proteins	*Culex quinquefasciatus*	–	–	Gut	Digestion	[146]
Aa-proteins	*Aedes albopictus*	–	–	Gut	Digestion	[149]
ISPL5; ISP13	*An. gambiae*	–	–	Gut	Immunity	[153, 154]
AgSP14D1	*An. gambiae*	–	–	Gut	Immunity	[155]
SP14A; SP14D2; SP18D; SP2A	*An. gambiae*	–	–	Haemolymph	Immunity	[156]
SP22D	*An. gambiae*	–	–	Haemolymph	Immunity	[157]
ClipSP 1; SerF 2; SerF 3	*Anopheles dirus*	–	–	Fat body	Immunity	[158]
Pptryp 1-4	*Phlebotomus papatasi*	BapNa; N-Suc-Ala-Ala-Pro-Phe-pNA	2-nitro-4-carboxyphenyl-N, N-diphenylcarbamate	Gut	Digestion	[164]
Lltryp 1; Lltryp 2	*Lutzomyia longipalpis*	–	TPCK; PMSF; TLCK	Gut	Digestion	[165, 166]
Chymotrypsin-like						
JHA15	*Ae. aegypti*	N-Suc-Ala-Ala-Pro-Phe-pNA	Chymostatin	Gut	Development	[147]
AaChymo	*Ae. aegypti*	–	–	Gut	Digestion	[139]
AaSP II-V	*Ae. aegypti*	–	–	Gut	Digestion	[139]
Aa-protein	*Ae. albopictus*	–	–	Gut	Digestion	[149]
Anchym 1; Anchym 2	*An. gambiae*	–	–	Gut	Digestion	[150]
AgChyL	*An. gambiae*	N-Suc-Ala-Ala-Pro-Phe-pNA	–	Gut	Digestion	[151]
Anachy1; Anachy2	*Anopheles aquasalis*	Z-Phe-Arg-AMC	PMSF; TLCK	Gut	Digestion	[152]
Andchy1; Andchy2	*Anopheles darlingi*	–	–	Gut	Digestion	[152]
Ppchym1; Ppchym 2	*P. papatasi*	BapNa; N-Suc-Ala-Ala-Pro-Phe-pNA	2-nitro-4-carboxyphenyl-N, N-diphenylcarbamate	Gut	Digestion	[164]
Colagenase-like						
AaSPI	*Ae. aegypti*	–	–	Gut	Immunity	[139]
Metallo; Leucine-aminopeptidases						
LpNa	*Ae. aegypti*	Leu-pNa	Leuhistin; Amastatin; Bestatin	Gut	Digestion	[159]
Carboxypeptidase A and B						
HPA; HA	*Ae. aegypti*	Hippuril-Phe; Hippuril-Arg	1–10 Phenanthroline	Gut	Digestion	[159]
MMP						
AgMMP 1	*An. gambiae*	Casein	–	Gut	Digestion; immunity	[160]

### In other dipterans

Four trypsin-like (Pptryp1-Pptryp4) and two chymotrypsin-like (Ppchym1 and Ppchym2) cDNAs from *Phlebotomus papatasi* midgut have been studied. Ppchym1 and Ppchym2 expression profiles are similar to *Ae. aegypti* early and late trypsins, suggesting that a two-phase digestive mechanism also occurs in sand flies [164]. In *Lutzomyia longipalpis* two trypsin-like SP cDNAs, Lltryp1 and Lltryp2, have also been described [165]. While Lltryp1 is expressed in females after a blood meal, Lltryp2 is detected in both sexes regardless of feeding. *L. longipalpis* infection by *Leishmania major* promastigotes has not modified the expression levels of Lltryp1 and Lltryp2. It is plausible that different results may be obtained when infections are performed with *Leishmania chagasi* amastigotes [166]. In contrast, a correlation has been reported between *Leishmania* spp. infections and reduced trypsin-like SP activity in *P. papatasi*. The presence of specific inhibitors of trypsin in the blood meal prevents the early killing of *L. major* and *Leishmania donovani* in the midgut of this insect species, implying that the proteolytic activity in the sand fly midgut modulates vector susceptibility to infections [167–169]. Finally, a cathepsin B (GmcathB) from tsetse *Glossina morsitans morsitans* is induced after blood meal and can degrade bovine Hb and serum albumin in a wide pH range [170].

## Conclusions

In this review, we discussed the role of some proteases involved in processes related to the blood-feeding habit of arthropods: blood sucking, digestion, yolk formation, immunity and in the transmission and survival of pathogens. The diversity and expansion of proteases families assigned to these processes in the insects and ticks presented here reinforce the hypothesis that haematophagy evolved independently several times in haematophagous organisms, including within Insecta [15, 171]. It is possible that to adapt to blood-feeding habit, the set of proteins available in an ancestral non-haematophagous lineage have faced the challenge of developing new biological activities, resulting in the generation of the essential nutrients and detoxification of the adverse molecules [172].

The combination of arthropod cysteine/aspartic protease network for protein digestion may have evolved early in metazoan, predating the transition to serine proteases based digestion found in insects [85, 173, 174]. The remarkable exception for this picture occurs in triatomine bugs, a feature consistent with its evolutionary path from hemipteran ancestors feeding on sap and seeds, which may have re-routed the extracellular digestion from trypsins to cathepsins to avoid the trypsin inhibitors found on seeds [4]. Therefore, two evolutionarily distinct haematophagous vectors, ticks and triatomines, utilize this combination of cysteine and aspartic proteases to perform most of the digestive function [11]. Notably, the evolutionary path of these vectors indicates that their conserved bulk of digestive proteases are related by convergent evolution [172].

We have noticed that authors have given different names for proteases belonging to the same clan or isoforms even in a single organism. This issue calls for a unified nomenclature to avoid misunderstanding and ambiguities. Thus, we suggest that a careful sequence analysis be done when describing a new gene to name it according to the clan (or clan prototype) name and in the case of already studied proteases, rename them. MEROPS database (merops.sanger.ac.uk) is very useful to define accurate systematic nomenclature, since it allows search for organism peptidase, family, clans, substrates, inhibitors and other resources.

Considering the central functions that proteases from haematophagous arthropod vectors have, a question that arose is whether proteases could be employed as potential targets for the development of alternative strategies for arthropod vector control, for instance, in the emerging investigation on arthropod protease inhibitors [175]. Another question would be if these molecules can stimulate immune mechanisms in the host that could block the transmission of microorganisms by these vectors. Resolving this issue would require the isolation of the best candidates and to accomplish extensive investigations aiming to determine the molecular interactions and mechanisms involved in the functions of proteases.

## Additional file

Additional file 1: Overview of multiple sequence alignment of ten predicted Cathepsin L from *R.* (*B.*) *microplus.* The alignments were performed by multiple sequence comparison using Clustal Omega (http://www.ebi.ac.uk/Tools/msa/clustalo/), and manually edited using Aline (http://bondxray.org/software/aline.html). Black, fully conserved residues. Gray, not conserved residues. Blue, catalytic residues. (BMP 2207 kb)

## Abbreviations

20-HE: 20-hydroxyecdysone
CUB: Complement C1r/C1s, Uegf, Bmp1 domain
DREDD: Caspase-8 like
EDNH: Egg development neurosecretory hormone
Hb: Haemoglobin
IMD: Immunodeficiency pathway
JH: Juvenile hormone
LDLa: Low-density lipoprotein receptor domain class A
PAR: Proteinase-activated G protein coupled receptors
PO: Phenoloxidase
SPs: Serine proteases
SUSHI: Domain abundant in complement control proteins
Vg: Vitellogenin
Vt: Vitellin
