# Supplementary figures and images for: Proteases of haematophagous arthropod vectors are involved in blood-feeding, yolk formation and immunity - a review
Source: Parasit Vectors. 2017 Feb 13;10:79. doi: 10.1186/s13071-017-2005-z (PMC5307778; doi:10.1186/s13071-017-2005-z)

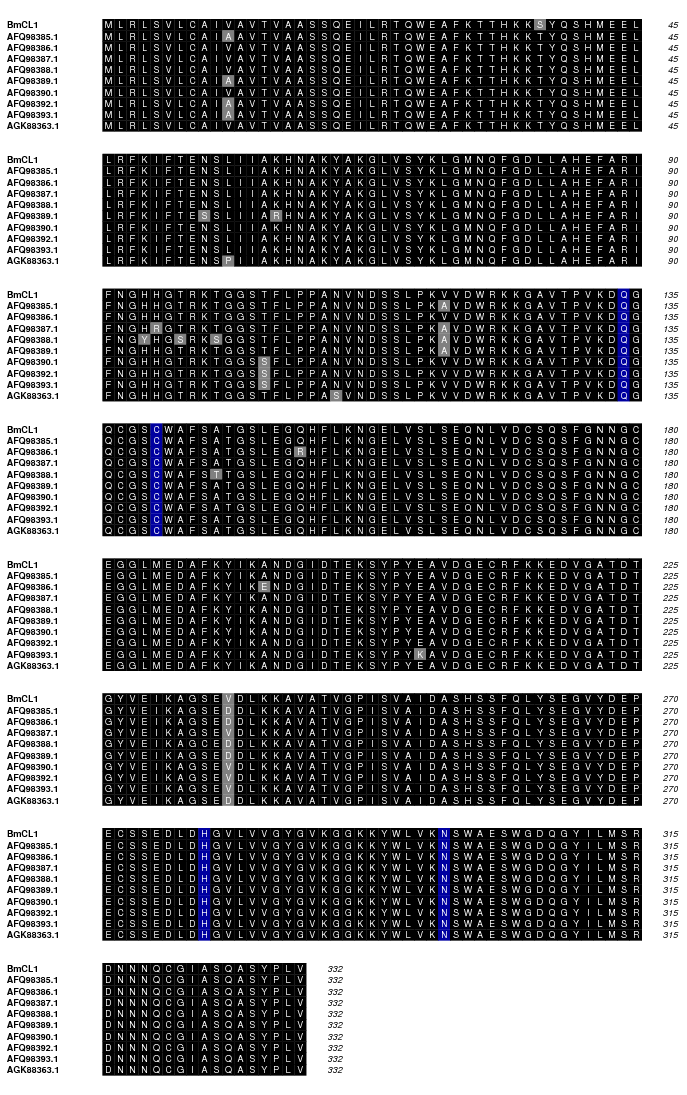

Supplement: Additional file 1: — Overview of multiple sequence alignment of ten predicted Cathepsin L from R. (B.) microplus. The alignments were performed by multiple sequence comparison using Clustal Omega (http://www.ebi.ac.uk/Tools/msa/clustalo/), and manually edited using Aline (http://bondxray.org/software/aline.html). Black, fully conserved residues. Gray, not conserved residues. Blue, catalytic residues. (BMP 2207 kb) [file 13071_2017_2005_MOESM1_ESM.bmp]
